# Identification of the Novel TMEM16A Inhibitor Dehydroandrographolide and Its Anticancer Activity on SW620 Cells

**DOI:** 10.1371/journal.pone.0144715

**Published:** 2015-12-11

**Authors:** Yujie Sui, Fei Wu, Junfeng Lv, Hongxia Li, Xin Li, Zhenwu Du, Meiyan Sun, Yuhao Zheng, Longfei Yang, Lili Zhong, Xingyi Zhang, Guizhen Zhang

**Affiliations:** 1 Key Laboratory for Molecular and Chemical Genetics of Critical Human Diseases of Jilin Province, Jilin University Bethune Second Hospital, Changchun, P. R. China; 2 Department of Gynecology and Obstetrics, Jilin University Bethune Second Hospital, Changchun, P. R. China; 3 Department of Radiology, Jilin University Bethune Second Hospital, Changchun, P. R. China; 4 Department of Dermatology, Jilin University Bethune First Hospital, Changchun, P. R. China; 5 Department of Thoracic Surgery, Jilin University Bethune Second Hospital, Changchun, P. R. China; Institute of Biochemistry and Biotechnology, TAIWAN

## Abstract

TMEM16A, a calcium-activated chloride channel (CaCC), is highly amplified and expressed in human cancers and is involved in the growth and metastasis of some malignancies. Inhibition of TMEM16A represents a novel pharmaceutical approach for the treatment of cancers and metastases. The purpose of this study is to identify a new TMEM16A inhibitor, investigate the effects of this inhibitor on the proliferation and metastasis of TMEM16A-amplified SW620 cells, and to elucidate the underlying molecular mechanism *in vitro*. We identified a novel small-molecule TMEM16A inhibitor dehydroandrographolide (DP). By using patch clamp electrophysiology, we showed that DP inhibited TMEM16A chloride currents in Fisher rat thyroid (FRT) cells that were transfected stably with human TMEM16A and in TMEM16A-overexpressed SW620 cells but did not alter cystic fibrosis transmembrane conductance regulator (CFTR) chloride currents. Further functional studies showed that DP suppressed the proliferation of SW620 cells in a dose- and time-dependent manner using MTT assays. Moreover, DP significantly inhibited migration and invasion of SW620 cells as detected by wound-healing and transwell assays. Further mechanistic study demonstrated that knockdown of human TMEM16A decreased the inhibitory effect of DP on the proliferation of SW620 cells and that TMEM16A-dependent cells (SW620 and HCT116) were more sensitive to DP than TMEM16A-independent cells (SW480 and HCT8). In addition, we found that treatment of SW620 cells with DP led to a decrease in TMEM16A protein levels but had no effect on TMEM16A mRNA levels. The current work reveals that DP, a novel TMEM16A inhibitor, exerts its anticancer activity on SW620 cells partly through a TMEM16A-dependent mechanism, which may introduce a new targeting approach for an antitumour therapy in TMEM16A-amplified cancers.

## Introduction

Multiple genetic abnormalities take place during the process of transformation of a normal cell into a cancer cell. Ion channels are involved in many of these processes such as proliferation, apoptosis, migration and invasion [1]. The pharmacologic blockade of ion channels is a promising antitumour therapy. Transmembrane protein 16A (TMEM16A) calcium-activated chloride channels (CaCCs) are amplified and highly expressed in several human cancers, such as head and neck squamous carcinoma (HNSCC), breast cancer, oesophageal squamous carcinoma (ESSC), gastrointestinal stromal tumours and prostate cancer [2,3,4,5,6,7,8]. TMEM16A is also known as ANO1, DOG1, TAOS2, and ORAOV2, because it was known to be amplified and overexpressed in cancers before it was identified as a CaCCs with eight putative transmembrane domains and N- and C-termini oriented towards the cytoplasm [9,10,11]. TMEM16A is expressed in various tissues, including the secretory epithelium, sensory and olfactory neurons and smooth muscle, and contributes to the regulation of epithelial fluid transport, saliva production, vascular smooth muscle contraction, and gut motility [3,11]. TMEM16A knock-out mice die soon after birth because of tracheomalacia [12]. TMEM16A is located on chromosome 11q13, which is one of the most frequently amplified regions in many types of human malignancies and is associated with poor prognosis in patients [13]. Overexpression of TMEM16A is associated with tumourigenesis and tumour growth and migration [3,14,15,16]. Down-regulation of TMEM16A protein levels by RNAi and pharmacologic blockade decrease the proliferation of breast cancer, prostate cancer and HNSCC by affecting the activation of the MAPK/AKT signalling pathways [14,17]. TMEM16A regulates the migration and metastasis of some types of cancers [18]. Therefore, TMEM16A may represent a promising target for cancer therapy, and inhibitors of TMEM16A have great potential for use as a therapeutic drug. However, currently, few TMEM16A inhibitors have been identified, and how TMEM16A inhibitors affect cancer progression and metastasis is unknown.

Here, using patch clamp electrophysiology, we characterize a novel small-molecule TMEM16A inhibitor, dehydroandrographolide (DP), which was isolated from Andrographis paniculata (Burm. F.) Nees (Chuan-xin-lian), and has been reported to possess multiple pharmacological activities, including anti-inflammation, anti-cancer, anti-bacterial, anti-virus and anti-hepatitis activity [19]. Other reports have also suggested that DP has hepatoprotective and anti-inflammatory properties [20,21]. However, its anti-cancer activity remains ambiguous.

Our previous studies have demonstrated that TMEM16A is highly amplified and overexpressed in the human colon cancer cell line SW620, and knockdown of TMEM16A inhibited the proliferation, migration and invasion ability of SW620 cells [16]. Therefore, in this study, we focused on the potential effects and molecular mechanism of DP on TMEM16A-dependent SW620 cells.

## Materials and Method

### Chemicals and solutions

DP was purchased from the National Institute for the Control of Pharmaceutical and Biological Products in China, and its purity was determined to be approximately 98% by HPLC measurement. DP was dissolved in dimethylsulfoxide (DMSO) to make a 100 mmol/L stock solution and diluted prior to experiments. Niflumic acid (NFA) was purchased from Sigma-Aldrich, and T16A_inh_-A01 was obtained from Alan Verkman (University of California, San Francisco, CA, USA)

### Cell culture

The cell lines SW620, SW480, HCT8, HCT116, and GES were purchased from the American Type Culture Collection (ATCC). SW480 and SW620 cells were grown in L15 Medium (Sigma, USA). HCT8 and HCT116 were grown in RPMI medium 1640 (Sigma, USA). MES were grown in Dulbecco’s Modified Eagle’s Medium (Sigma, USA). Fisher rat thyroid (FRT) cells were transfected stably with human TMEM16A, and FRT cells coexpressing human CFTR and YFP-H148Q/I152 L were obtained from Alan Verkman (University of California, San Francisco, CA, USA) and were cultured in Coon’s modified F12 medium [22]. All media were supplemented with 10% foetal bovine serum, 100 U/ml penicillin and 100 μg/ml streptomycin. Cells were incubated at 37°C in 5% CO_2_ and 95% air.

### Patch clamp electrophysiology

Cells were seeded on glass coverslips, and patch clamp recordings were performed using an EPC10 amplifier (HEKA, Lambrecht/Pfalz, Germany) in combination with Patchmaster (HEKA Lambrecht/Pfalz, Germany) at room temperature. Pipettes were prepared from borosilicate capillary glass using a micropipette puller (PC-10, NARISHIGE, Japan) and then fire-polished with a microforge (MF-900, NARISHIGE, Japan). The resistance of the pipettes in the bath solution was 2–5 MΩ. Cells were perfused with a bath solution containing 145 mM NaCl, 5 mM KCl, 2 mM MgCl_2_, 1 mM CaCl_2_, 20 mM sucrose, 5 mM glucose and 5 mM HEPES (pH 7.4 with NaOH). Whole-cell recordings were performed on TMEM16A-expressing FRT cells, as reported [16]. The pipette solution contained 146 mM N-methyl-D-glucamine chloride (NMDG-Cl), 2 mM MgCl_2_, 5 mM EGTA and 8 mM HEPES (pH 7.4 with NMDG). 600 nM free Ca^2+^ was added to activate TMEM16A as described previously [16]. Whole cell currents were elicited by applying voltage from a holding potential of 0 mV to voltages between −100 to +100 mV at 20 mV increments. Inside-out recordings were performed on CFTR-expressing FRT and SW620 cells. The CFTR channels in CFTR-expressing FRT cells were first activated with 2.75 mM ATP and 25 U/ml PKA until the current reached steady state. All test solutions contained 10 U/ml PKA and 1 mM ATP to maintain the phosphorylation level. DP and CFTR_inh_-172 were separately added to affect the CFTR. For the SW620 cells, the pipette solution contained 140 mM NMDG-Cl, 2 mM MgCl_2_, 5 mM CaCl_2_ and 10 mM HEPES (pH 7.4 with NMDG) and the perfusion solution contained 150 mM NMDG-Cl, 2 mM MgCl_2_, 10 mM EGTA, 8 mM Tris and 10 mM HEPES (pH 7.4 with NMDG). Free Ca^2+^ (1 μM) was added to activate TMEM16A, and then DP, NFA and T16A_inh_-A01 were added separately to inhibit TMEM16A. After the seal resistance reached > 40 GΩ, the membrane was excised and the membrane potential was maintained at −50 mV. The currents were filtered at 100 Hz with an eight-pole Bessel filter (LPF-8, Warner Instruments, LLC, Hamden, CT, USA) and digitized using a computer at a sampling rate of 500 Hz. All inside-out patch experiments were performed using a fast solution exchange perfusion system (SF-77B, Warner Instruments). The dead time of the solution change was 30 ms, and data analysis was performed for whole-cell and inside-out recordings using the Igor software as described previously [23].

### Assessment of cell morphology and viability

Cells (2 × 10^4^ cells/ml) in the absence or presence of DP at different concentration were incubated for 24 h in 24-well tissue culture plates. At the end of the incubation period, the culture medium was removed and cells were washed with phosphate-buffered saline (PBS pH 7.4) and the cells were photographed under an inverted microscope ((Olympus, Tokyo, Japan) at 100 × magnification.

The cell viability was measured using the MTT assay as previously described [16]. Briefly, 5000 cells were seeded in a 96-well plate and incubated with DP at various concentrations for 24 h and 48 h. The MTT reagent (500 μg/mL) was then added to each well, and the cells were further incubated for 4 h. Subsequently, 150 μl DMSO was added to dissolve the formazan crystals. The number of viable cells was quantified using the absorbance measured at 570 nm with a microplate reader (Thermo Scientific, USA).

### Knockdown of TMEM16A expression in SW620 cells

TMEM16A/ANO1 siRNA and scrambled siRNA were synthesized by GeneChem Co., Ltd. (Shanghai, China). The two sites for siRNA targeting of the human TMEM16A/ANO1 gene (GenBank NM_018043) were #1, CGTGTACAAAGG CCAAGTA (1077–1095 nt) and #2, CGAAGAAGATGTACCA CAT (837–855 nt). RNA interference was conducted according to the manufacturer’s instructions.

### Wound-healing assay

Cells were grown to a confluent monolayer in a 6-well plate, scraped with a sterile, 200-μl tip and washed twice with PBS. After incubation with L15 medium containing 1% foetal bovine serum following treatment with 5 μM DP or DMSO, the cells were photographed at 0, 24, 48 or 72 h under an inverted microscope (Olympus, Tokyo, Japan) at 40 × magnification. These experiments were carried out in triplicate. The distances between the wound edges were measured with a graduated ruler, and the relative scratch breadth was determined by the ratio of the average breadth of the treatment cells versus the average breadth of the control cells.

### Cell invasion assay

*In vitro* cancer cell migration and invasion activities were evaluated in transwells as described previously [16]. Cells were treated with 5 μM DP or DMSO. For cell migration, 1 × 10^5^ cells in 200 μl of medium with 1% FBS were seeded in the upper transwell insert chamber containing a polycarbonate filter (6.5-mm diameter, 8-μm pores; Corning Costar, Corning, NY, USA). L15 medium (600 μl) with 10% FBS (chemoattractant) was added to the lower chamber, and the plates were incubated for 72 h at 37°C in 5% CO_2_. To analyze cell invasion, the transwell insert chambers were coated with Matrigel. The cells that did not migrate were removed from the top of the transwell filters by scraping. The cells that had penetrated the Matrigel were fixed with paraformaldehyde, stained with Coomassie blue and counted under an inverted microscope (100 × magnification). The number of penetrated cells represented the migration activity.

### Western blotting

Proteins from the cell lysates were prepared from cell lines as previously described [16]. Equal amounts of protein were denatured and separated by SDS-PAGE, transferred onto PVDF membranes, and incubated with polyclonal antibodies to TMEM16A (Abcam, ab53212; 1:100) and mouse β-actin antibody (Cell Signalling; 1:500). The peak intensity of each band was visualized using an Enhanced Chemiluminescence kit (Amersham, Little Chalfont, UK) on X-ray film (Millipore Corporation, Billerica, USA).

### QT-PCR

Total RNA was extracted from cells using the TRIzol reagent (Invitrogen, Carlsbad, CA, USA) according to the manufacturer’s introductions. The concentration, purity and integrity of the RNA were measured using a NanoDrop2000 spectrophotometer (Thermo Scientific). The RNA (500 ng) was reverse transcribed to cDNA using reverse transcriptase with the PrimeScriptTM RT Reagent Kit (TaKaRa). Quantitative, real-time PCR was performed using SYBR Green chemistry on a Roche LightCycler^®^ 480 System. The PCR primers of human origin used for real-time PCR were as follows: TMEM16A-sense 5’-GCGTCCACATCATCAACATC-3’ and TMEM16A-antisense 5’-ATCCTCGTGGTAGTCCATCG-3. TMEM16A expression levels were normalized to the reference gene β-actin.

### Statistical analysis

Statistical analysis was performed using SPSS statistics (version 17.0) with a two-tailed Student’s *t*-test. A *p* < 0.05 was considered to be statistically significant. The data are expressed as the mean ± *SD*.

## Results

### The effects of DP on TMEM16A-CaCC activity in FRT cells transfected with human TMEM16A

**Fig 1:**
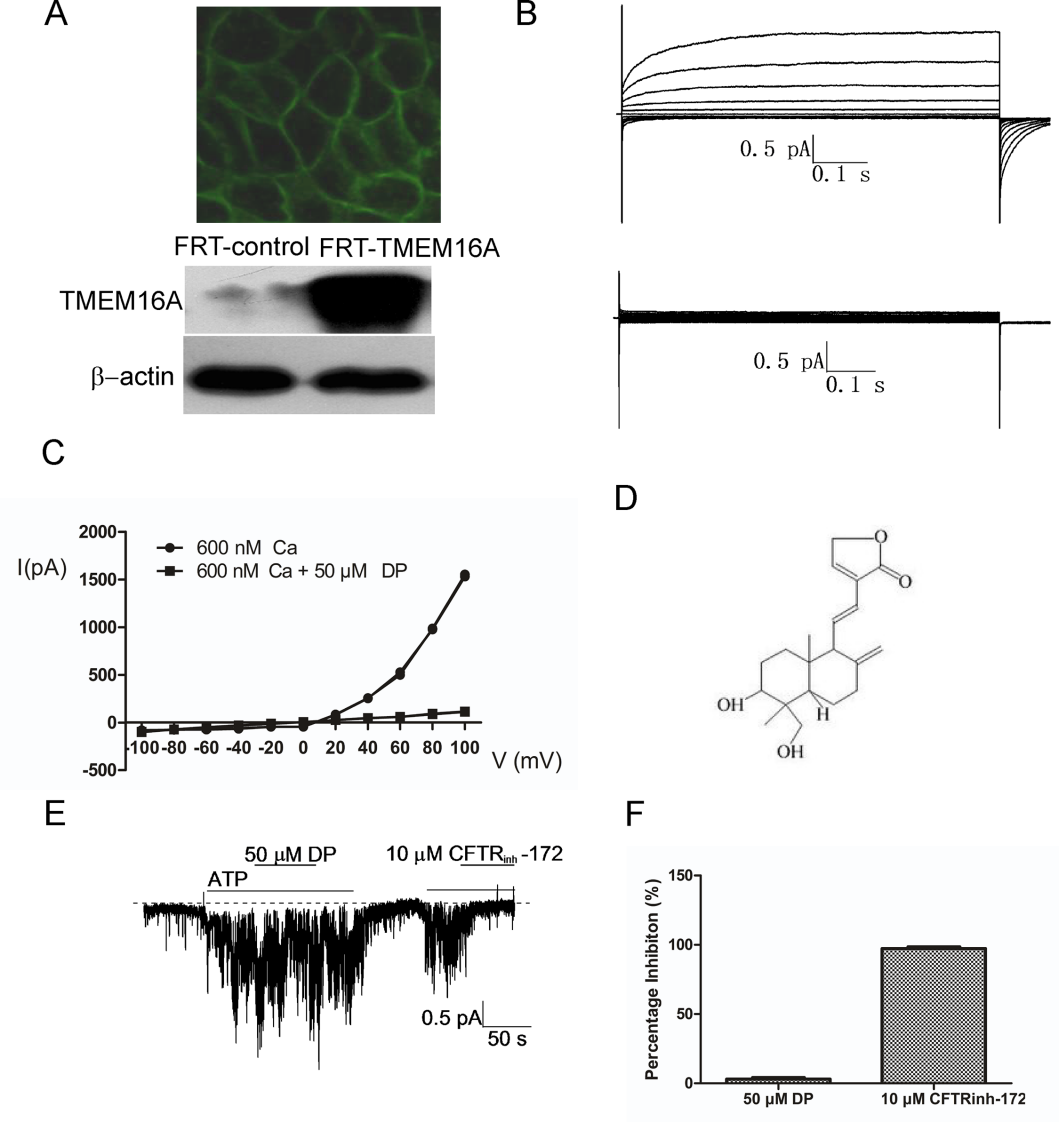
Identification of a small molecule inhibitor (DP) of human TMEM16A. A, FRT cells stably transfected with human TMEM16A and GFP showing green membrane fluorescence (above) and TMEM16A protein by western blotting (below). B, Examples of whole-cell currents recorded from FRT-TMEM16A cells at a holding potential of 0 mV, followed by pulsing voltages between ±100 mV in steps of 20 mV in the absence (above) or presence of 50 μM DP (below). TMEM16A CaCC currents were elicited by 600 nM of free calcium in pipette solution. C, Current/voltage (I/V) plot of the mean currents at the middle of each voltage pulse. D, Chemical structure of DP. E, CFTR Cl^-^ current trace recorded from CFTR-expressing FRT cells. CFTR Cl^-^ current was elicited by 1 mM ATP, followed by the addition of DP and CFTR_inh_-172. The dashed line represents zero current. F, The bars represent the percentage inhibition of DP (n = 6) and CFTR_inh_-172 (n = 4) on CFTR Cl^-^ current.

Using patch clamp electrophysiology, we identified a novel TMEM16A inhibitor, DP, which inhibits CaCC currents in FRT cells that are stably transfected with human TMEM16A-GFP. Fig 1A shows green GFP fluorescence on the membrane of TMEM16A-transfected FRT cells and confirms the protein expression of human TMEM16A in FRT-TMEM16A cells. Whole-cell patch clamp recording demonstrated that 50 μM DP caused an almost complete inhibition of the TMEM16A chloride current induced by 600 nM free Ca^2+^ (Fig 1B). Moreover, DP had an inhibitory role at all voltages (Fig 1C), indicating that it employed a voltage-independent mechanism of inhibition. Fig 1D shows the chemical structure of DP, which is chemically unrelated to previously reported CaCC inhibitors. To study the Cl^-^ channel selectivity of DP inhibition, we further tested the activities of DP on the CFTR Cl^-^ channels using inside-out patch clamp. Fig 1E shows that 50 μM DP had no obvious effects on CFTR currents, even though CFTR_inh_-172 almost completely blocked CFTR currents.

### The effects of DP on TMEM16A-CaCC activity in SW620 cells

In a previous study, we found that TMEM16A was highly amplified and overexpressed in the human colon cancer cell line SW620 and RNAi-mediated knockdown of TMEM16A in SW620 cells decreased cell proliferation, migration and invasion. DP, a Terpene isolated from Andrographis paniculata Nees, has been reported to possess a broad range of pharmacological effects, including anti-cancer activities. Therefore, in the present study, we investigated the potential effects of DP on TMEM16A-dependent SW620 cells and further examined whether TMEM16A was involved in the DP-induced regulation of activity in SW620 cells.

**Fig 2:**
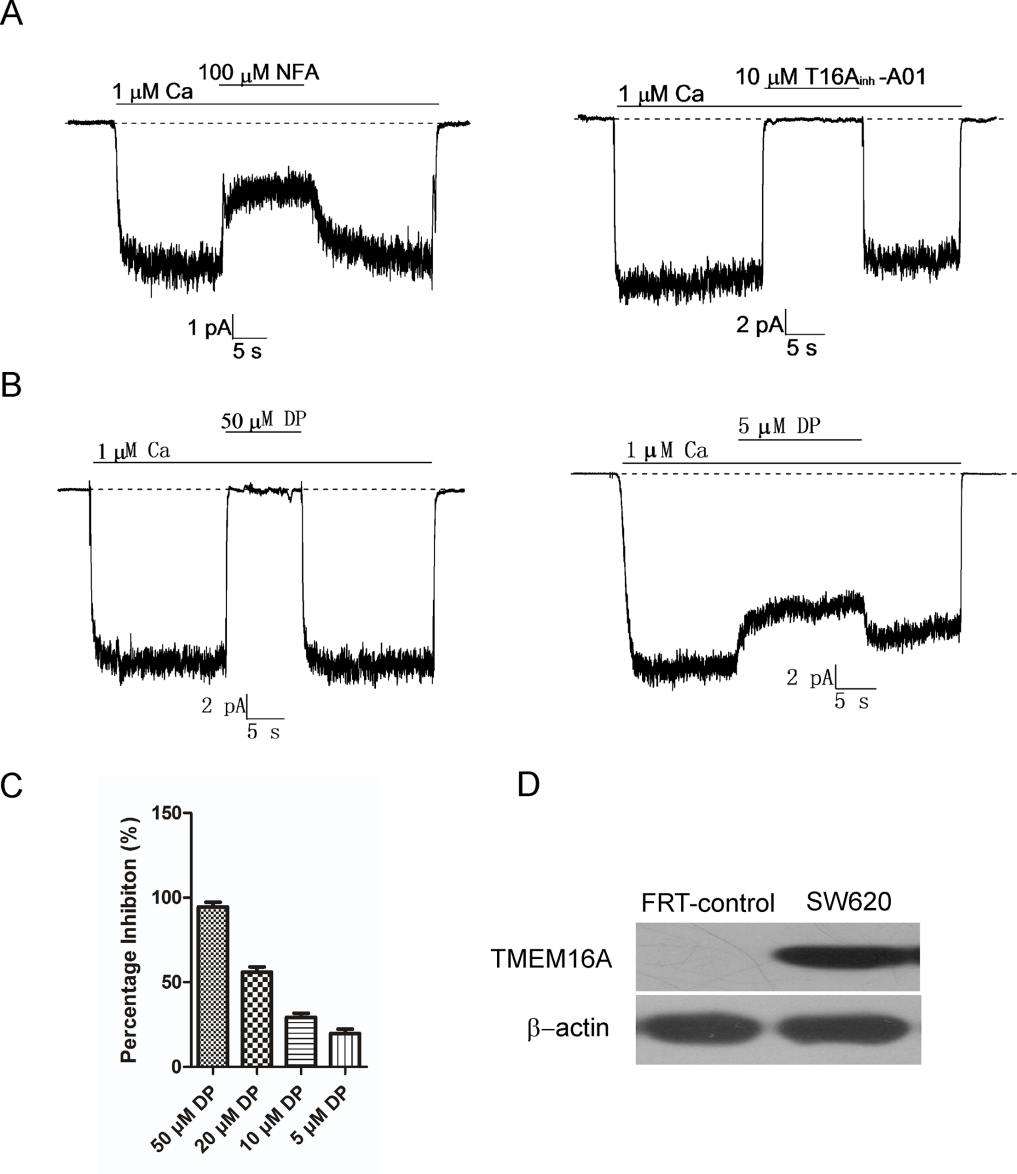
The inhibitory effect of DP on TMEM16A-CaCC activity in SW620 cells. A, TMEM16A-CaCC currents from SW620 cells were confirmed by 100 μM NFA (left) and 10 μM T16A_inh_-A01 (right). B, Representative traces from inside-out patches showing inhibition of TMEM16A-CaCC by 50 μM DP (left) and 5 μM DP (right). C, Summary of the percent of inhibition for 50 μM (n = 8), 20 μM (n = 3), 10 μM (n = 4) and 5 μM DP (n = 4). D, Immunoblot of TMEM16A protein in SW620 cells. Null FRT cells serves as negative control.

To explore the effects of DP on TMEM16A-CaCC activity in SW620 cells, we performed inside-out patch clamp recordings on SW620 cells using a 1 μM free calcium pipette solution to activate TMEM16A-CaCC currents. Above all, the currents activated by 1 μM Ca^2+^ from patches of SW620 cells were confirmed to be TMEM16A-CaCC currents by inhibition of the chloride channel inhibitor (NFA) and the TMEM16A specific inhibitor T16A_inh_-A01 (Fig 2A). Then, the effects of DP on TMEM16A-CaCC activity in SW620 cells were detected. As Fig 2B shows, CaCC currents in TMEM16A-expressing SW620 cells were attenuated rapidly after the addition of DP and restored quickly after DP washout. The effects of DP on TMEM16A-CaCC currents were dose-dependent. Addition of 50 μM DP resulted in almost complete inhibition, and 5 μM DP reduced CaCC currents by 19.6 ± 4.5% (Fig 2C). Repeated application of DP elicited similar reductions in the amplitude of the current.

### The effect of DP on TMEM16A-dependent SW620 cell growth

A recent study declared that the inhibition of TMEM16A-CaCC activity alone is not sufficient to inhibit TMEM16A-dependent cell proliferation [24]. They demonstrated that some TMEM16A inhibitors, such as CaCC_inh-A01_, displayed inhibitory effects on cell proliferation in TMEM16A-amplified cell lines and some TMEM16A inhibitors, such as T16A_inh-A01_, did not. Therefore, in this study, we will further explore the effect of DP on the growth of TMEM16A-amplified SW620 cells.

**Fig 3:**
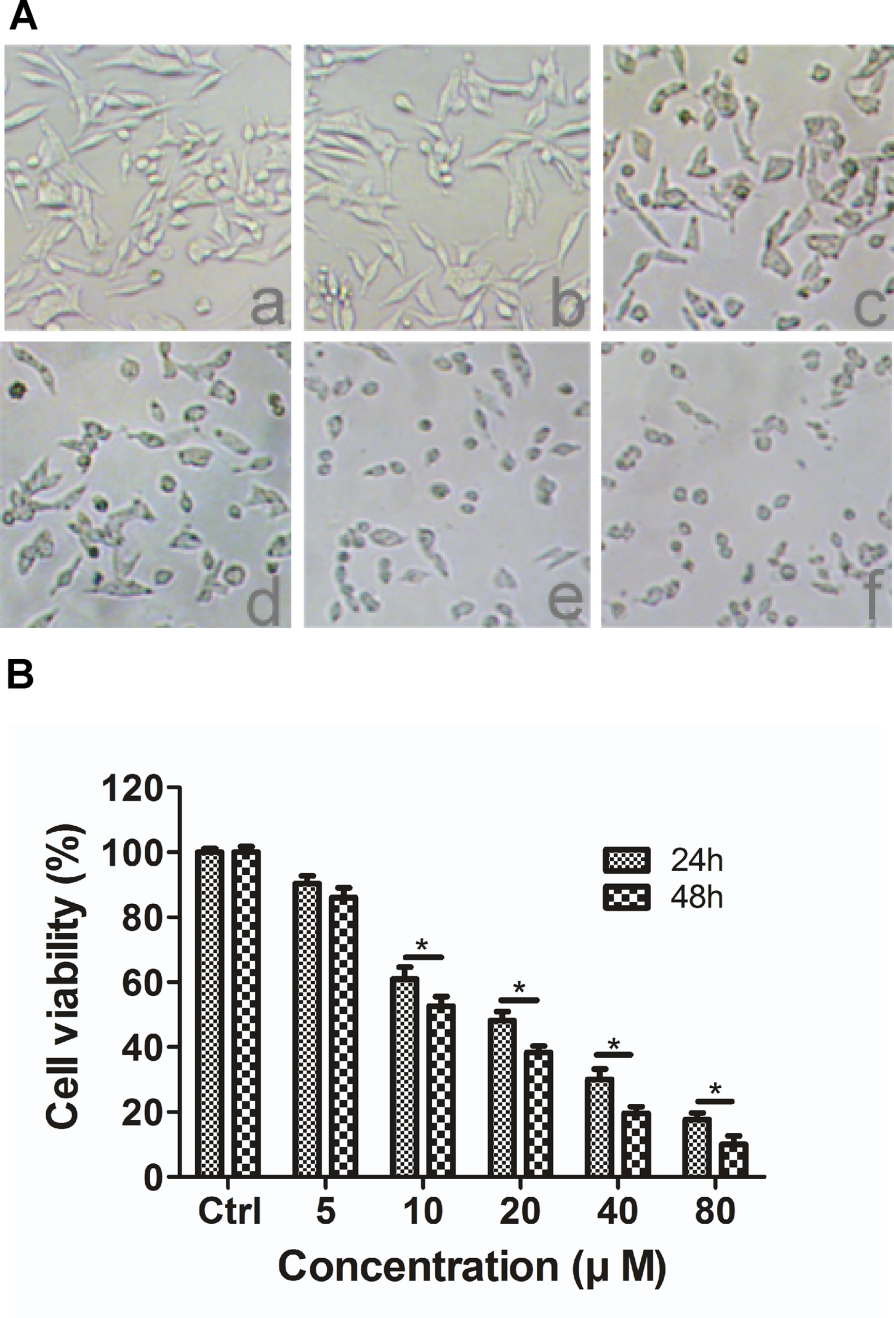
The effect of DP on the morphological characteristics and the viability of SW620 cells. A, Morphological changes of SW620 cells observed under phase-contrast microscopy (100 × magnification) after treating cells with (a) control, (b) 5 μM, (c) 10 μM, (d) 20 μM, (e) 40 μM or (f) 80 μM of DP for 24 h. B, SW620 cells were treated with various concentrations (0, 5, 10, 20 40 and 80 μM) of DP for 24 or 48 h, as described in the methods. Cell viability was measured using the MTT assay. The results are expressed as the mean ± S.D. (n = 3); * p < 0.05.

To assess the effects of DP on the growth of TMEM16A-amplified SW620 cells, we treated SW620 cells with 5, 10, 20, 40 or 80 μM DP for 24 h and captured images under an inverted microscope. These images show that DP induced morphological changes in cells and displayed cytotoxicity compared to the control group (Fig 3A). Moreover, cell viability was tested by MTT assay after SW620 cells were incubated with different concentrations of DP for 24 h and 48 h. The results show that DP inhibited the proliferation of SW620 cells in a dose- and time-dependent manner (Fig 3B).

### The effect of DP on the motility of SW620 cells

Our previous study found that knockdown of TMEM16A in SW620 cells not only decreased cell proliferation but also inhibited migration and invasion. Therefore, we further detect the effect of DP on migration and invasion of SW620 cells. In view of growth experiment results, DP at a concentration of 5 μM did not significantly affect the viability of SW620 cells. Thus, we chose concentration of 5 μM for subsequent motility and invasion experiments.

**Fig 4:**
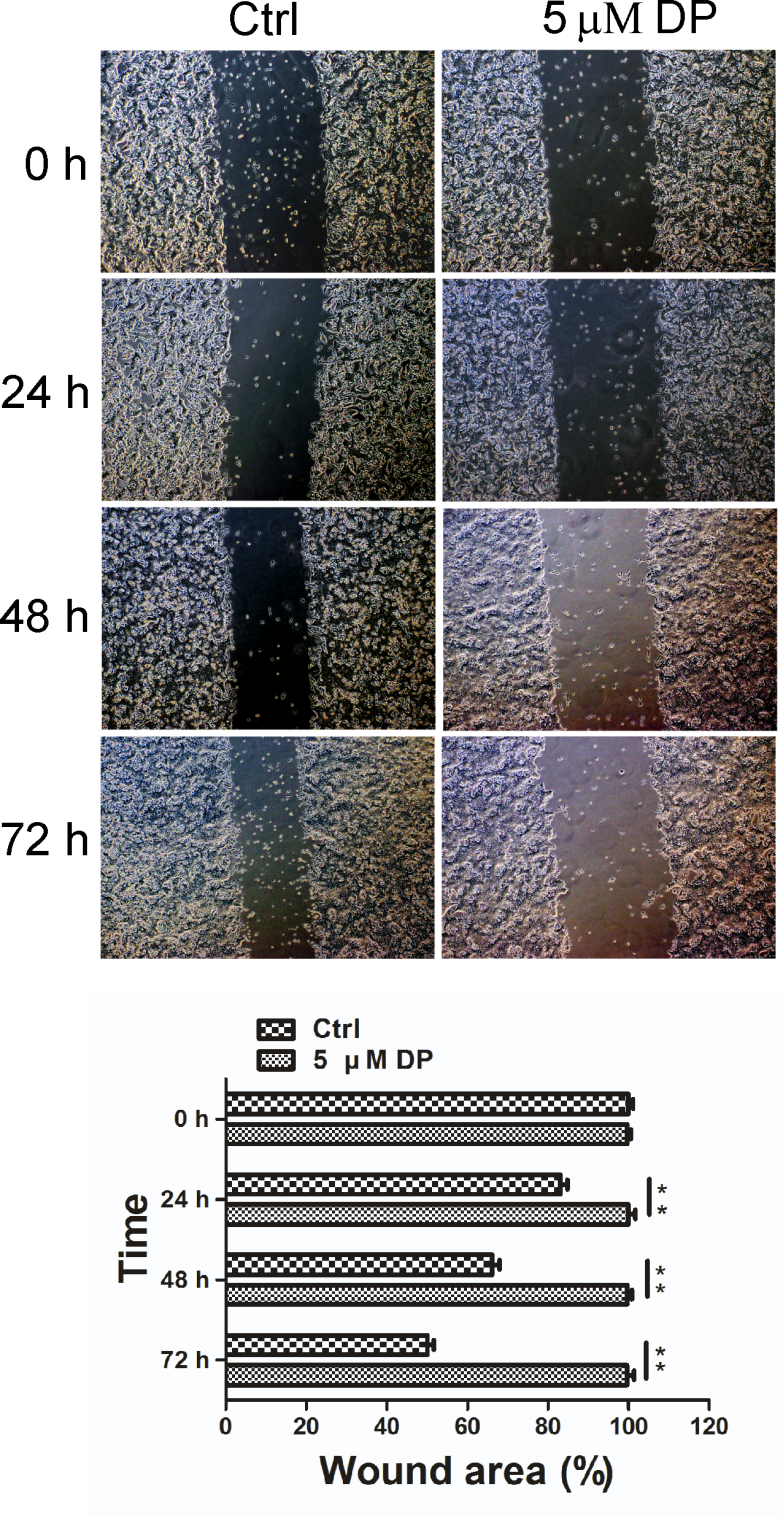
Effects of DP on SW620 cell motility. Migration of SW620 cells was assessed by a wound-healing assay in the presence of 5 μM DP, compared to the control group. Representative images of wound closure were taken at 0, 24, 48 and 72 h after injury under 40 × magnification (above). Bar graphs of wound area are shown (below). Values are the means ± SD; n = 3; ** p < 0.01. Ctrl, control.

The effect of DP on the movement of TMEM16A-dependent SW620 cells was determined by wound-healing assay. As shown in Fig 4, after SW620 cells were treated with DMSO (control) or DP (5 μM) for 24, 48 or 72 h, the wound areas were observed and captured under a light microscope. The results revealed that the migration of the cells was almost completely perturbed after the application of 5 μM DP for 24 h compared to the untreated control cells.

### The effect of DP on the invasion of SW620 cells

**Fig 5:**
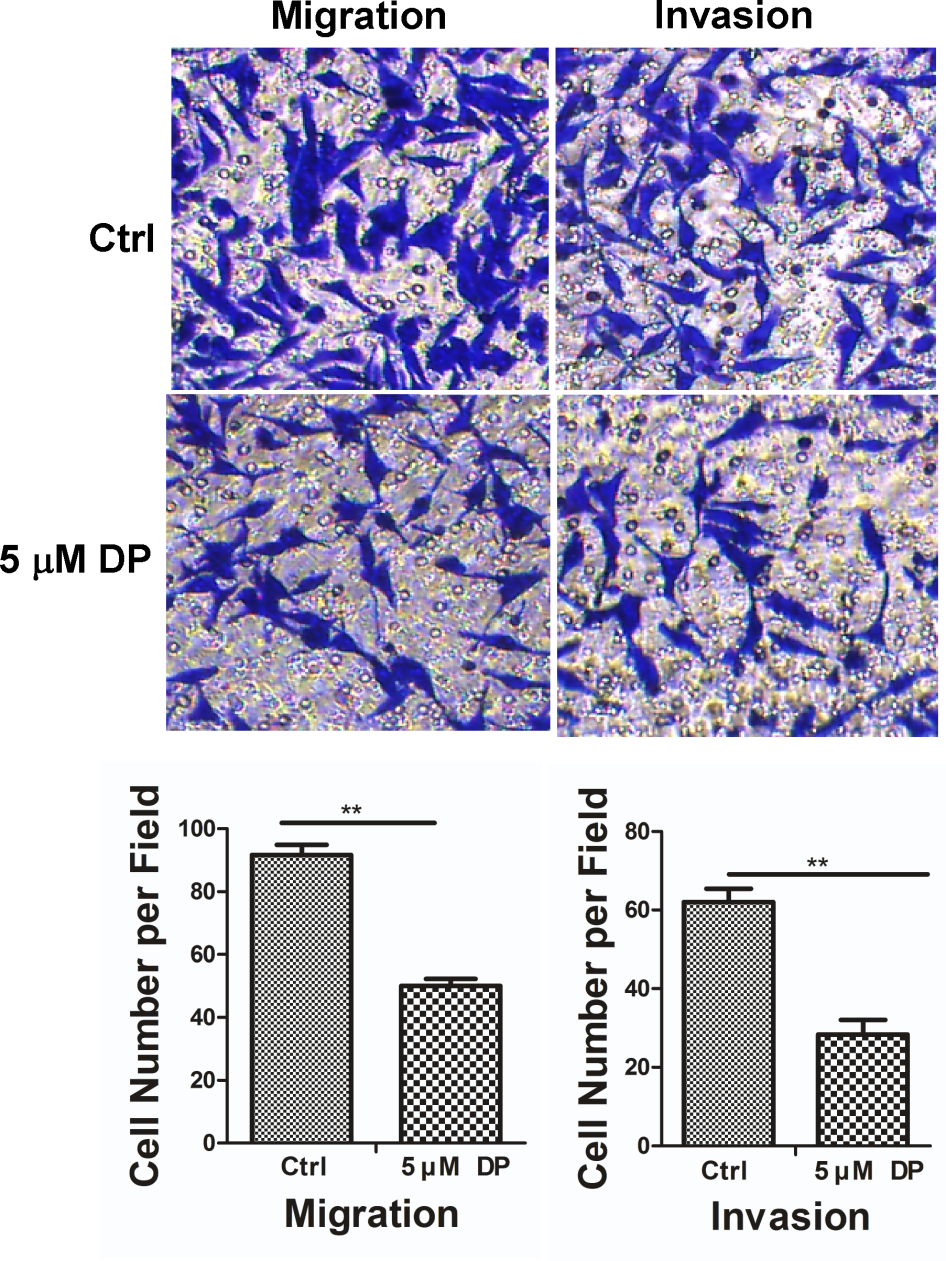
Effects of DP on SW620 cell migration and invasion *in vitro*. Migration (without Matrigel) and invasion (with Matrigel) of SW620 cells are significantly suppressed by 5 μM DP compared with the control group through transwell penetration assays. Non-migrated cells were scraped with a cotton swab. Cells that penetrated the transwell filters were stained with Coomassie blue. Representative images are shown (above). Bar graphs showed cells number per field of SW620 cell migration (lower left) or invasion (lower right). All data are shown as the mean ± SD. n = 3; ** p < 0.01. Ctrl, control.

To explore the effect of DP on the invasive ability of SW620 cells, we performed transwell migration and invasion assays in the absence or presence of 5 μM DP. As Fig 5 shows, in the transwell migration assay, the number of cells treated with 5 μM DP for 72 h that penetrated the membrane largely decreased compared with the control group. Similarly, a comparable effect of 5 μM DP on the invasive ability of SW620 cells was also observed in a parallel invasion assay. These data indicated that SW620 cells exposed to 5 μM DP have reduced migration and invasion abilities.

### The role of TMEM16A in the DP-induced inhibition of SW620 cell proliferation

**Fig 6:**
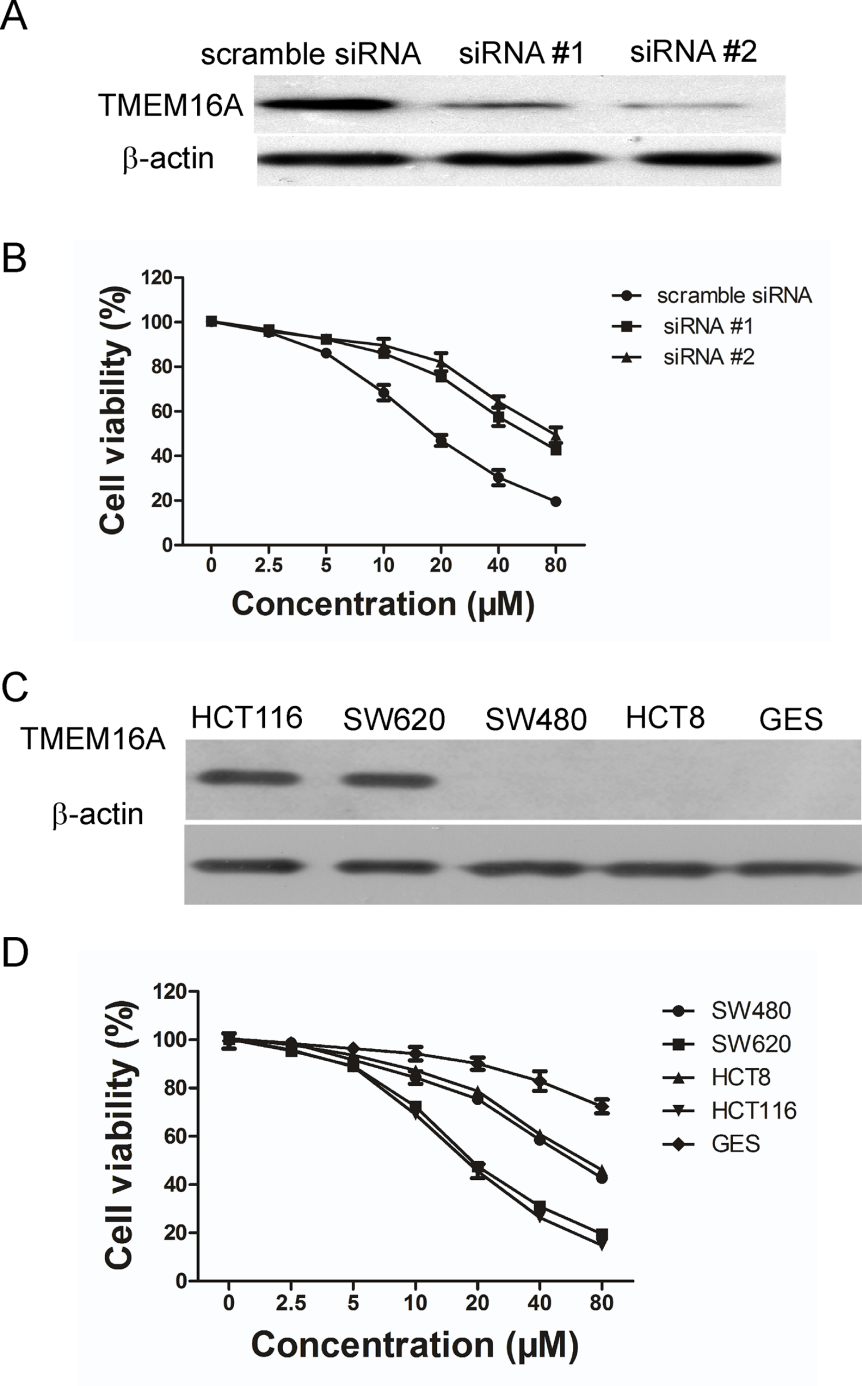
Involvement of TMEM16A in DP-induced growth inhibition. A, Shown here is a western blot analysis of TMEM16A protein expression in SW620 cells that were transfected with siRNA #1 and #2. B, Effect of DP on SW620 cells after TMEM16A was knocked down, compared to scrambled siRNA. The viability of cells was determined by an MTT assay. Data are expressed as the mean ± SD (n = 5). DMSO served as the solvent control. C, TMEM16A expression was examined in the indicated cell lines by western blotting. Representative blots are shown. D, Effect of DP on growth of the indicated cell lines (mean ± SD, n = 4).

To investigate whether TMEM16A is involved in the DP-induced growth inhibition of TMEM16A-dependent SW620 cells, we transfected two TMEM16A siRNA sequences (#1 and #2) into SW620 cells to silence the expression of TMEM16A. As shown in Fig 6A, immunoblotting of cell lysates with TMEM16A antibody revealed a marked decrease in TMEM16A expression in TMEM16A siRNA SW620 cells compared to scramble siRNA SW620 cells. Quantification of the immunoblots showed that TMEM16A siRNA #1 caused a 73.5% reduction in TMEM16A protein expression and TMEM16A siRNA #2 caused a 82.1% reduction. Compared to the scramble siRNA group, DP displayed a less inhibitory effect on SW620 cell proliferation in TMEM16A siRNA groups, although the DP phenotype is not completely reversed (Fig 6B). This result suggested that DP inhibited the growth of SW620 cells only partially, via a TMEM16A-dependent mechanism.

To examine the toxic effect of DP, we observed the effect of DP on nontumor cells GES (SV40 immortalized gastric epithelium cell) and found that GES cells were slightly affected by DP (Fig 6D). Next, we compared the effect of DP on cell growth between TMEM16A-dependent (HCT116 and SW620) and TMEM16A-independent cell lines (HCT8 and SW480). Consistent with TMEM16A being the target of DP, TMEM16A-dependent cells were found to be more sensitive to DP than TMEM16A-independent cells (Fig 6D). The results further indicated that the inhibition of proliferation in SW620 cells by DP is partially TMEM16A-mediated.

### The role of TMEM16A in the DP-induced inhibition of SW620 cell migration and invasion

**Fig 7:**
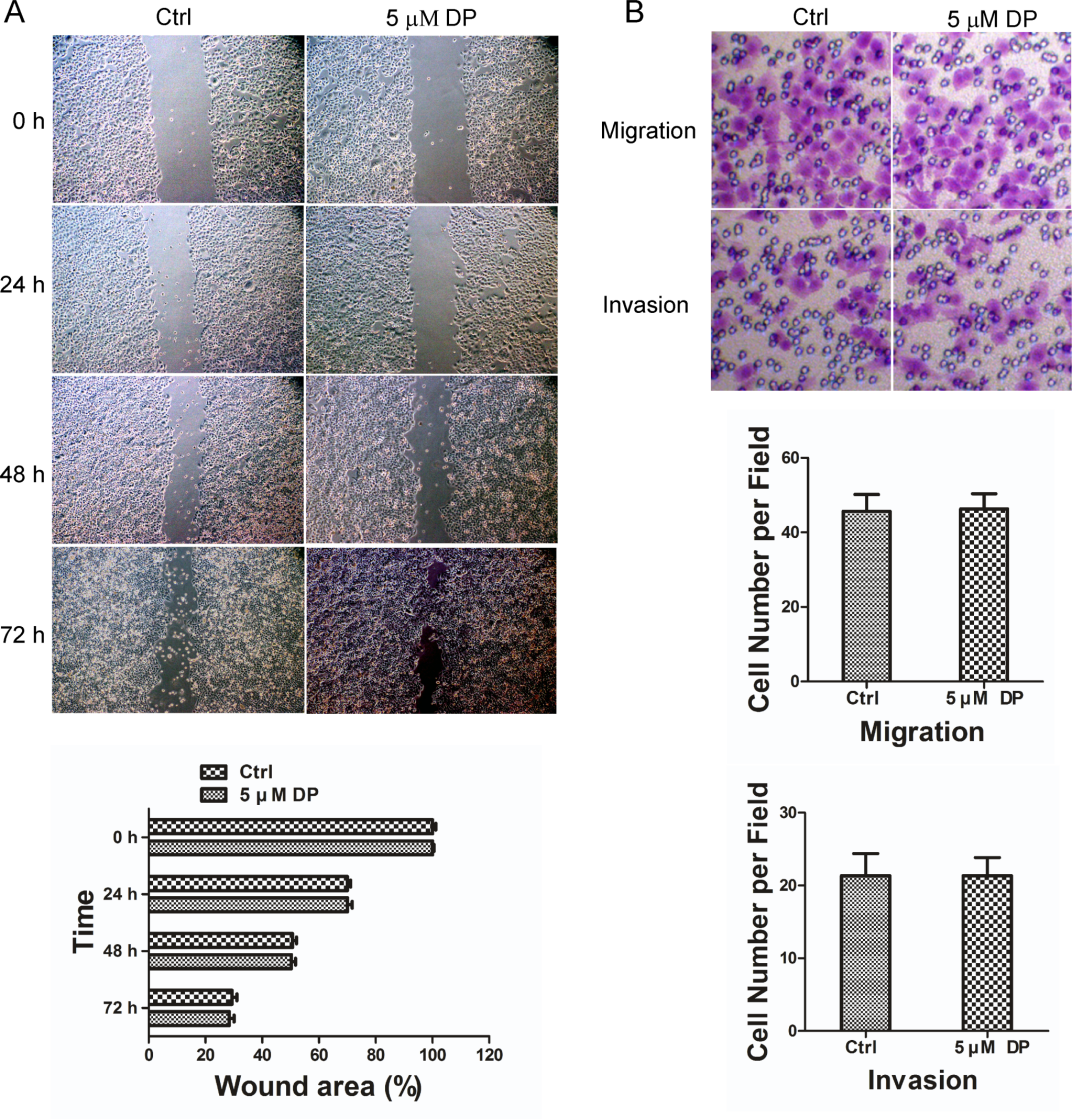
Effect of DP on SW480 cell migration and invasion *in vitro*. A, Wound-healing assay showed that 5 μM DP did not affect movement of SW480 cells. Representative images of wound closure were taken at 0, 24, 48 and 72 h after injury under 40 × magnification (above). Bar graphs of upper panel are shown (below). Values are the means ± SD; n = 3. Ctrl, control. B, Transwell penetration assays showed that 5 μM DP did not affect migration (without Matrigel) and invasion (with Matrigel) of SW480 cells compared with the control group (above). Bar graphs showed the cell number per field of SW480 cell migration (middle) or invasion (below). Values are the means ± SD; n = 3; Ctrl, control.

To elucidate the potential role of TMEM16A in the DP-induced decrease in SW620 cell migration and invasion, we chose SW480 cells as the control, which shares the same genetic background as SW620 cells but lacks amplified TMEM16A. We detected the effect of DP on the migration and invasion of SW480 cells using wound-healing and transwell invasion assays. As Fig 7 shows, the application of 5 μM DP has no detectable effect on the migration and invasion behaviour of SW480 cells compared to the control group. These results showed that TMEM16A-dependent SW620 cells were sensitive to DP compared to TMEM16A-independent SW480 cells, which may be due to the involvement of TMEM16A.

### The effect of DP on TMEM16A expression in SW620 cells

**Fig 8:**
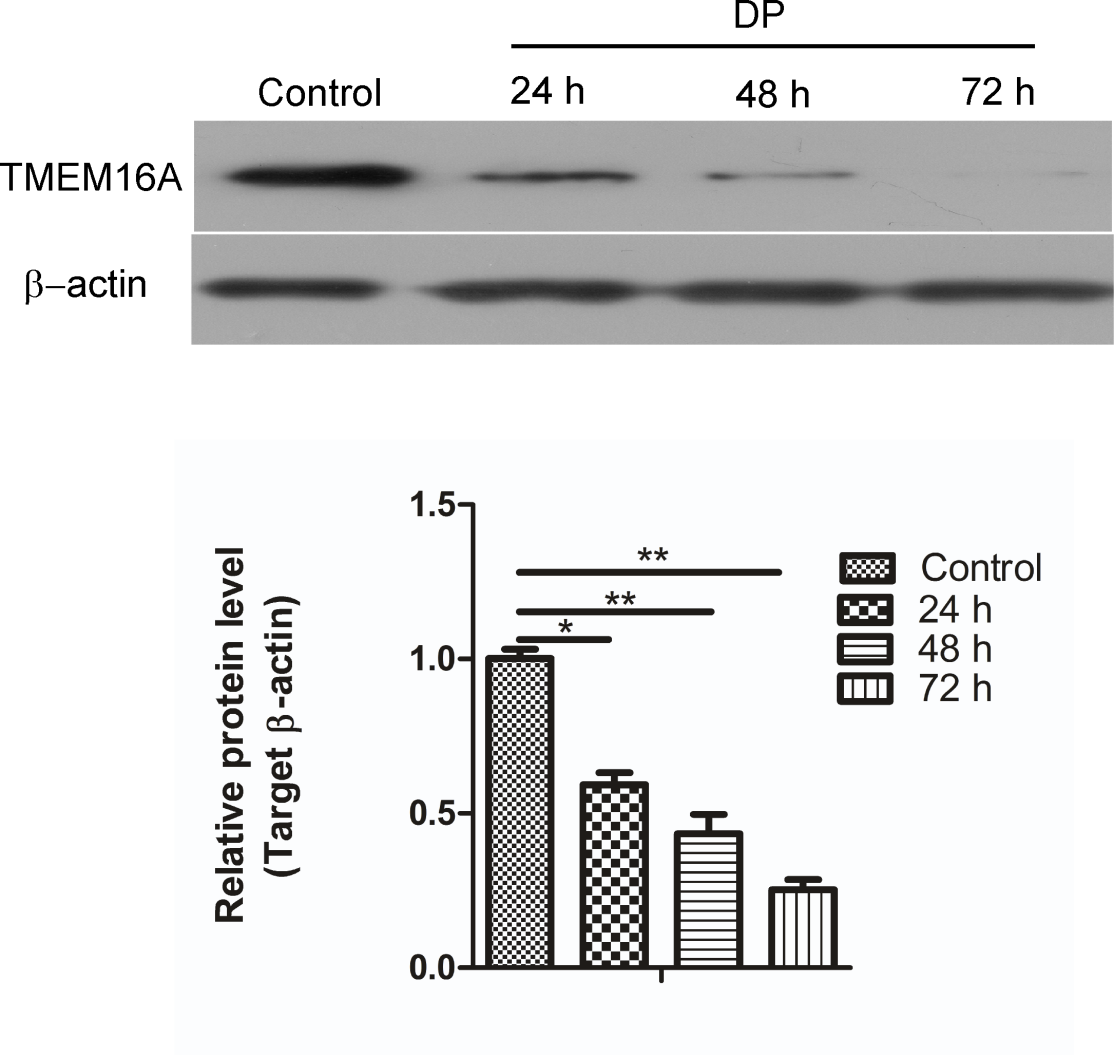
Effects of DP on TMEM16A expression. TMEM16A protein expression were determined by western blotting after SW620 cells were treated with 5 μM of DP for 24, 48 or 72 h (above). Control SW620 cells were treated with DMSO for 24 h. β-actin was used as a loading control. Representative western blots are shown. The bar graph summarizes the relative expression level of TMEM16A protein (below). Expression of TMEM16A protein was normalized with the expression levels of β-actin. All data are shown as the mean ± SD. n = 3; * p < 0.05, ** p < 0.01.

To further confirm the contribution of TMEM16A in the inhibition of migration and the invasion by DP, TMEM16A mRNA and protein expression were evaluated using real-time quantitative PCR and western blotting after the SW620 cells were treated with DP over a time course. As shown in Fig 8, DP significantly decreased the protein expression level of TMEM16A in a time-dependent manner compared with the control. However, the treatment of SW620 cells with DP had no effect on TMEM16A mRNA levels (data not shown). These results indicate that DP might regulate TMEM16A expression at the post-transcriptional level in SW620 cells.

## Discussion

In the present study, we identified the novel TMEM16A channel inhibitor DP, which could inhibit CaCC currents from FRT cells that were stably transfected with human TMEM16A, by using patch clamp electrophysiology. It has been reported that TMEM16A channels play an important role in epithelial Cl^-^ secretion, smooth muscle contraction, olfactory signal transduction and cancer progression [25]. Many inhibitors of TMEM16A-CaCCs are non-selective Cl^-^ channel inhibitors that inhibit both CaCCs and CFTR [26]. Here we found that 50 μM DP completely inhibited TMEM16A currents but did not alter CFTR currents, indicating that DP exhibits selectivity for TMEM16A channels. However, the specificity of DP on TMEM16A channels needs to be further established, for example, whether DP affects other Cl^-^ channel currents, such as CLCs and bestrophin-1, needs to be investigated. Because TMEM16A channel inhibitors that are useful in treating disease are rare, this finding is important. It is possible that DP or its molecular derivatives may be developed to treat some diseases related to TMEM16A, such as excessive mucus, hypertension, pain, diarrhoea, and cancer.

Andrographolide (AP), DP, and new andrographolide (NP) are three components of *Andrographis paniculata* Nees, which is an important herbal medicine that is widely used in China, India and other Southeastern Asian countries. AP has been reported to inhibit cancer cell proliferation, induce cell-cycle arrest and promote apoptosis in human cancer cells [27,28]. However, the effects of DP on cancer cells are unknown. In a previous study, we found that TMEM16A is amplified and highly expressed in highly metastatic SW620 cells and knockdown of TMEM16A in SW620 cells decreased metastasis [16]. A recent study found that some TMEM16A inhibitors such as CaCC_inh_-A01 could inhibit both TMEM16A-CaCC activity and proliferation in TMEM16A-amplified cell lines, but other TMEM16A inhibitors, such as T16A_inh_-A01 did not inhibit proliferation of TMEM16A-dependent cell lines, although they have been reported to inhibit TMEM16A-CaCC activity [24]. Therefore, after identifying DP as a new TMEM16A inhibitor, we investigated whether DP has effects on TMEM16A-amplified SW620 cells. This study is the first report of DP not only inhibiting TMEM16A CaCC currents but also significantly blocking the growth, migration and invasion of SW620 cells. Our data demonstrate for the first time that DP possesses potent anti-cancer activity in TMEM16A-expressing cells. Its effect on metastasis is greatly anticipated for colon cancer treatment.

To determine the role of TMEM16A on the regulation of DP-induced growth inhibition of TMEM16A-overexpressing SW620 cells, we first decreased the expression of TMEM16A by transfecting siRNA sequences into SW620 cells and observed a change in the DP phenotype. As a result, we found that DP displayed a decreasing inhibitory effect on the proliferation of SW620 cells in siRNA groups compared to the scrambled group, although the effects of DP were not reversed completely. Subsequently, we compared the effects of DP on the growth of TMEM16A-dependent cell lines (HCT116 and SW620) and TMEM16A-independent cell lines (HCT8 and SW480) and found that DP was relatively ineffective in TMEM16A-independent cells. These results indicated that the DP-induced inhibitory effects on the growth of SW620 cells are partially TMEM16A-mediated.

We further investigated the possible role of TMEM16A in the DP-mediated migration and invasion of SW620 cells. As the control we chose SW480 cells, which have the same genetic background, but lack TMEM16A expression. Consistent with our anticipation that TMEM16A may act as the target of action for DP, TMEM16A-independent SW480 cells were found to be almost no response to DP in migration and invasion experiments. This phenomenon could be explained by the fact that DP affects the migration of SW620 cells by regulating TMEM16A, which plays a crucial role in supporting migration [16,29,30]. Further data demonstrated that DP led to a decrease in TMEM16A protein levels but had no effect on mRNA levels in SW620 cells. These results suggested that TMEM16A is at least partially involved in DP-induced inhibition of cell migration and invasion.

DP at the concentration of 5 μM showed inhibitory effect on cell migration and invasion, but not obvious effect on the viability of SW620 cells. These results may indicate that DP affected cell proliferation and invasion by different underlying mechanism. There is still a possibility that other pathways may be involved in these processes, and further studies need to be carried out to reveal the detailed signalling mechanism involved in DP activity

As far as we know, this is the first study to report that DP inhibits the growth and invasion of metastatic colorectal cancer cells in vitro and to reveal its possible mechanism. Nonetheless, our findings provide the new insight that DP may act, in part, through the inhibition of TMEM16A expression and may support TMEM16A as a novel target for antitumour therapy in TMEM16A-amplified cancers.
